# Diphlorethohydroxycarmalol Attenuates Methylglyoxal-Induced Oxidative Stress and Advanced Glycation End Product Formation in Human Kidney Cells

**DOI:** 10.1155/2018/3654095

**Published:** 2018-04-22

**Authors:** Seon-Heui Cha, Yongha Hwang, Soo-Jin Heo, Hee-Sook Jun

**Affiliations:** ^1^College of Pharmacy, Gachon University, Incheon 21936, Republic of Korea; ^2^Lee Gil Ya Cancer and Diabetes Institute, Gachon University, Incheon 21936, Republic of Korea; ^3^Gachon Medical and Convergence Institute, Gachon Gil Medical Center, Incheon 21565, Republic of Korea; ^4^Jeju International Marine Science Center for Research & Education, Korea Institute of Ocean Science & Technology (KIOST), Jeju 63349, Republic of Korea

## Abstract

Diabetic nephropathy is the leading cause of end-stage renal disease in patients with diabetes mellitus. Oxidative stress has been shown to play an important role in pathogeneses of renal damage in diabetic patients. Here, we investigated the protective effect of diphlorethohydroxycarmalol (DPHC), which is a polyphenol isolated from an edible seaweed, *Ishige okamurae*, on methylglyoxal-induced oxidative stress in HEK cells, a human embryonic kidney cell line. DPHC treatment inhibited methylglyoxal- (MGO-) induced cytotoxicity and ROS production. DPHC activated the Nrf2 transcription factor and increased the mRNA expression of antioxidant and detoxification enzymes, consequently reducing MGO-induced advanced glycation end product formation. In addition, DPHC increased glyoxalase-1 mRNA expression and attenuated MGO-induced advanced glycation end product formation in HEK cells. These results suggest that DPHC possesses a protective activity against MGO-induced cytotoxicity in human kidney cells by preventing oxidative stress and advanced glycation end product formation. Therefore, it could be used as a potential therapeutic agent for the prevention of diabetic nephropathy.

## 1. Introduction

Hyperglycemia is associated with protein glycation, and advanced glycation end products (AGEs) are generated by irreversible nonenzymatic reactions between carbohydrates and proteins. In diabetes, AGEs are increased in the extracellular matrix [1, 2] and can undergo autooxidation to generate other reactive intermediates, thereby resulting in diabetes development [3] and complications such as diabetic nephropathy (DN) [4, 5].

DN occurs in 20% to 40% of patients with diabetes and is the most likely cause of end-stage renal disease reported worldwide [6]. Although numerous factors contributing to the development of diabetic complications including DN have been proposed, AGEs are thought to be of major importance [7]. In particular, the accumulation of AGEs during the early prediabetic stages of disease may be involved in mechanisms contributing to the onset of type 2 diabetes, namely, insulin resistance and beta-cell damage [8]. Therefore, inhibition of AGE formation is considered to be one promising approach for the prevention and treatment of DN.

Recently, antiglycation has been considered as an effective strategy to slow down human senescence and disease development [9–11]. Therefore, much attention has been focused on finding AGE inhibitors, including searching natural compounds such as polyphenols [12–14].

Diphlorethohydroxycarmalol (DPHC) is a polyphenolic compound from the edible seaweed, *Ishige okamurae*. Several studies have shown various biological functions of DPHC including antioxidant activity [15, 16], antiadipogenic activity [17, 18], and extracellular matrix regulation [19]. The aim of this study is to determine the possible protective effect of DPHC against the formation of AGEs in human kidney (HEK) cells in response to methylglyoxal (MGO), a highly reactive carbonyl metabolite of glucose and a major precursor of AGEs.

## 2. Materials and Methods

### 2.1. Preparation of DPHC from *Ishige okamurae*

**Figure 1:**
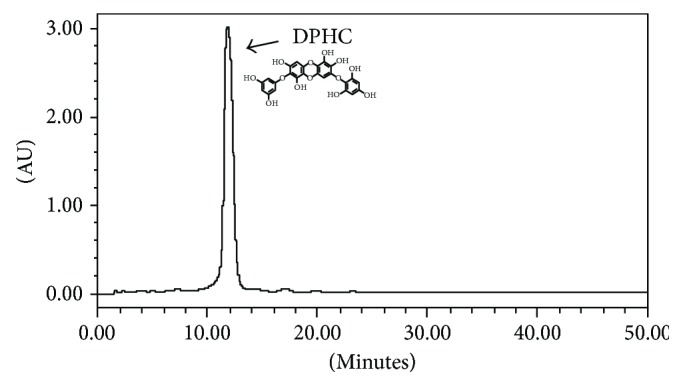
HPLC chromatogram of DPHC isolated from *Ishige okamurae*.

The brown seaweed, *Ishige okamurae*, was collected along the coast of Jeju Island, Korea, between October 2014 and March 2015. The samples were washed three times with tap water to remove the salt, epiphytes, and sand attached to the surface, followed by careful rinsing with freshwater, and maintained in a medical refrigerator at −20°C. Thereafter, the frozen samples were lyophilized and homogenized using a grinder prior to extraction. DPHC was isolated as previously described [20]. Briefly, the diethyl ether fraction of *I. okamurae* crude extract was subjected to silica gel (Sigma, St. Louis, MO) and Sephadex LH-20 column (GE Healthcare, England) chromatography and DPHC was purified by reversed-phase HPLC. The chemical structure and HPLC chromatogram of DPHC are presented in Figure 1. The purity of DPHC was >97%, based on the peak area of component absorbed at a specific wavelength in HPLC analysis.

### 2.2. Cell Culture

A human embryonic kidney cell line, HEK cells, was obtained from the American Type Culture Collection (ATCC, Manassas, VA). The cells were cultured in DMEM (WelGENE, Korea) supplemented with 10% FBS (WelGENE, Korea), 100 U/ml penicillin, and 100 *μ*g/ml streptomycin (WelGENE, Korea) and were maintained in a humidified incubator with 5% CO_2_. In all experiments except viability, cells were incubated in 40 *μ*M DPHC prior to the addition of 1 mM MGO (Alfa Aesar, England). Dimethyl sulfoxide (DMSO, Duchefa Biochemie, Netherlands) was used for the DPHC vehicle and PBS (WelGENE, Korea) used for the MGO vehicle.

### 2.3. Assessment of Cytotoxicity

Cell viability was estimated using a cell counting kit (CCK-8; Dojindo Laboratory, Japan) that measures mitochondrial dehydrogenase activity. For the CCK-8 assay, HEK cells (5 × 10^4^ cells/well) were seeded onto 96-well plates. After 16 h, the cells were treated with DPHC and/or MGO. We treated the cells with different concentrations of MGO (0.25, 0.5, 1, and 2 mM) for cell viability for 24 h and DPHC (20, 40, and 80 *μ*M) for 24 h for toxicity and chose the concentrations of 1 mM of MGO, which showed 50% cell viability, and 40 *μ*M of DPHC, which showed the best proliferative effects, for further study. To study the protective effects of DPHC, cells were pretreated with vehicle (control) or 40 *μ*M DPHC for 1 h and subsequently incubated with or without 1 mM MGO for 24 h at 37°C. The CCK-8 solution was then added to the wells to a total reaction volume of 110 *μ*l. After 2 h of the incubation, the absorbance was measured at a wavelength of 450 nm. The optical density of the formazan generated in the control cells was considered to represent 100% viability.

### 2.4. Estimation of the Intracellular Reactive Oxygen Species (ROS) Levels

HEK cells (1 × 10^5^ cells/well) were seeded onto 96-well plates. The cells were treated with vehicle (control) or 40 *μ*M DPHC, and 1 h later, 1 mM MGO was added and the cells were incubated for 24 h. Intracellular ROS production was detected by means of an oxidation-sensitive fluorescent probe dye, 2,7-dichlorofluorescein diacetate, which is deacetylated intracellularly and further oxidized to the highly fluorescent compound, dichlorofluorescein, in the presence of cellular peroxides [21]. After changing the media, 5 *μ*g/ml 2,7-dichlorofluorescein diacetate (Invitrogen, Carlsbad, CA) was added and cells were incubated for 30 min at 37°C. The fluorescence image was observed using a fluorescence microscope (Zeiss, Germany). To quantitatively evaluate the fluorescent images, the RGB image was analyzed by ImageJ software (https://imagej.nih.gov/ij/) and the mean value was used to obtain the bar graph.

### 2.5. Estimation of Intracellular Alkyl Radical Spectrum

HEK cells (2 × 10^5^ cells/well) were seeded onto 48-well plates. The cells were treated with vehicle (control) or 40 *μ*M DPHC, and 1 h later, 1 mM MGO was added and the cells were incubated for 24 h. The cells were dissociated with trypsin and resuspended in PBS. Intracellular alkyl radical was detected by the electron spin resonance spectrum. The dissociated cells were mixed with 10 mM 4-*α*-(4-pyridyl N-oxide)-N-tert-butylnitrone (Sigma, St. Louis, MO), incubated for 30 min at 37°C in a water bath, and then transferred to Teflon capillary tubes. The spin adduct was recorded using a JES-FA ESR spectrometer (JEOL Ltd., Japan) under the following measurement conditions: central field 3475 G, modulation frequency 100 kHz, modulation amplitude 2 G, microwave power 10 mW, gain 6.3 × 105, and temperature 25°C.

### 2.6. Quantitative Real-Time PCR

Total RNA was extracted from cells using RNAiso Plus (Takara Bio Inc., Japan), and cDNA was prepared using the PrimeScript™ cDNA synthesis kit (Takara Bio Inc., Japan) according to the manufacturer's instructions. cDNA samples were analyzed by the SYBR® Premix Ex Taq™, ROX plus (Takara Bio Inc., Japan) on Bio-Rad cyclers (Hercules, CA). Gene expression was normalized to the endogenous housekeeping control gene, cyclophilin, which was not influenced by DPHC or MGO. Relative expression was calculated for each gene using the ΔΔ*C*_T_ (where *C*_T_ is the threshold cycle) method. Statistical analysis of PCR data was based on duplicate samples. The primer sequences used are listed in Table 1.

### 2.7. Western Blotting

HEK cells (4 × 10^6^ cells/well) were seeded onto 6-well plates, and the cells were incubated with vehicle (control) or 40 *μ*M DPHC for 1 h and then further incubated with or without 1 mM MGO for 24 h. The cells were lysed using 1% Triton X-100-PBS and protease inhibitor cocktail (GenDEPOT, Barker, TX) for 20 min on ice. The lysates were fractionated by centrifugation at 12,000 rpm for 20 min at 4°C. The supernatant was used as the cytosolic fraction, and the pellets were used as the nuclear fraction for Western blotting. The protein concentrations were measured using a DC protein assay kit (Bio-Rad, Hercules, CA). The lysates were separated by SDS-PAGE and transferred to PVDF membranes (Millipore, Billerica, MA). Membranes were incubated with 5% skimmed milk for 1 h at room temperature and then incubated with primary antibodies overnight at 4°C. After washing extensively, membranes were incubated with the horseradish peroxidase-conjugated secondary antibody (Jackson ImmunoResearch, West Grove, PA). The signal was detected using WESTSAVE (AbFrontier, Korea) and the enhanced chemiluminescence system. ImageJ software was used to quantify the band intensity of Western blot. Primary antibodies used were anti-Nrf2 (Santa Cruz Biotechnology, Santa Cruz, CA) and anti-lamin B (Santa Cruz Biotechnology, Santa Cruz, CA).

### 2.8. Transfection and Image Analysis

The Nfr2-enhanced green fluorescent protein (EGFP) plasmid (pcDNA3-EGFP-C4-Nrf2) was purchased from Addgene (Cambridge, MA) [22]. siRNAs for human Nrf2, Glo-1, and control were purchased from Bioneer (Daejeon, Korea). HEK cells (5 × 10^4^ cells/well) were cultured on coverslips or in 60 mm cell culture dishes (1.5 × 10^5^ cells/dish), and 16 h later, cells were transfected using Lipofectamine 2000 (Invitrogen, Carlsbad, CA) or Lipofectamine RNAiMAX (Invitrogen, Carlsbad, CA) according to the manufacturer's instructions. For plasmid, after 3 h of transfection, the cells were incubated with vehicle (control) or 40 *μ*M DPHC for a further 33 h. For siRNA, after 36 h of transfection, the cells were incubated with vehicle (control) or 40 *μ*M DPHC for 1 h and then further incubated with 1 mM MGO for 24 h. The coverslips were washed twice with PBS and fixed in 4% paraformaldehyde for 15 min at room temperature. The fixed cells were then washed with PBS, blocked with PBS containing 1% BSA and 0.1% Triton X-100 for 30 min at room temperature, stained with DAPI (Invitrogen, Carlsbad, CA), mounted with VECTASHIELD (Vector Laboratories, Burlingame, CA), and observed with a confocal microscope (Zeiss, Germany). To evaluate Nrf2, five random fields were selected in each experiment and 5–10 cells were imaged in each field.

### 2.9. Immunohistochemistry

HEK cells cultured on coverslips were incubated with vehicle (control) or 40 *μ*M DPHC for 24 h. HEK cells cultured on coverslips were incubated with vehicle (control) or 40 *μ*M DPHC for 1 h, after which 1 mM MGO was added (Alfa Aesar, England). Six or 24 h later, the coverslips were washed twice with PBS and fixed in 4% paraformaldehyde for 15 min at room temperature. The fixed cells were then washed with PBS, blocked with PBS containing 1% BSA and 0.1% Triton X-100 for 30 min at room temperature, and incubated overnight with anti-Nrf2 (Santa Cruz Biotechnology, Santa Cruz, CA) at 4°C. Cells were then stained with a fluorescence-conjugated secondary antibody (Life Technologies, Carlsbad, CA) for 2 h, mounted with VECTASHIELD (Vector Laboratories, Burlingame, CA), and observed under a confocal microscope (Zeiss, Germany). To evaluate Nrf2, five random fields were selected in each experiment and 8–15 cells were imaged in each field. To evaluate cytosol and nucleus Nrf2, ImageJ (https://imagej.nih.gov/ij/) was used to quantify the fluorescence intensity.

### 2.10. Flow Cytometry Analysis

HEK cells (4 × 10^6^ cells/well) were seeded on 6-well plates. The cells were incubated with vehicle (control) or 40 *μ*M DPHC for 1 h and then further incubated with or without 1 mM MGO for 24 h. The cells were washed with PBS, 5 *μ*g/ml of 2,7-dichlorodihydrofluorescein diacetate (DCFH-DA, Invitrogen, San Diego, CA) was added, and the cells were incubated for 10 min at 37°C. The cells were fixed with 3% neutral buffered formalin (NBF, Sigma, St. Louis, MO) for 2 min at room temperature, and then NBF was removed and the cells were resuspended in PBS. DCF fluorescence intensity was detected by flow cytometry (FACS LSR Ц, BD Biosciences, Franklin Lakes, NJ). The representative median fluorescence intensity was overlaid using FlowJo software (Ashland, OR).

### 2.11. Determination of AGEs

HEK cells (4 × 10^6^ cells/well) were seeded onto 6-well plates, and the cells were incubated with vehicle (control) or 40 *μ*M DPHC for 1 h and then further incubated with or without 1 mM MGO for 24 h or 20 mM glucose for 30 h. The cells were incubated overnight in chloroform and methanol (2 : 1 *v*/*v*) mixture followed by homogenization in 0.1 N NaOH and centrifugation at 16,000 rpm for 15 min at 4°C. The supernatant was analyzed for AGE content at an excitation/emission wavelength of 370/440 nm against 0.1 N NaOH blank on a spectrofluorometer (Victor 3, Molecular Devices, San Jose, CA). 0.1 mg/ml of BSA (bovine serum albumin, Sigma, St. Louis, MO) preparation in 0.1 N NaOH was used as a reference (arbitrary units/mg protein).

### 2.12. Protein Carbonyl Content

HEK cells (4 × 10^6^ cells/well) were seeded onto 6-well plates, and the cells were incubated with vehicle (control) or 40 *μ*M DPHC for 1 h and then further incubated with or without 1 mM MGO for 24 h or 20 mM glucose for 30 h. Carbonylation of protein causing oxidative damage was measured in HEK cells using a Protein Carbonyl Content Assay Kit (Abcam, Cambridge, UK) according to the manufacturer's instructions.

### 2.13. Statistical Analysis

For statistical analysis, one-way ANOVA (more than three samples) or Student's *t*-test (two samples) was applied. Data are presented as means ± SEM. Values of *p* < 0.05 were considered significant.

## 3. Results

### 3.1. DPHC Attenuates MGO-Induced Cytotoxicity

**Figure 2:**
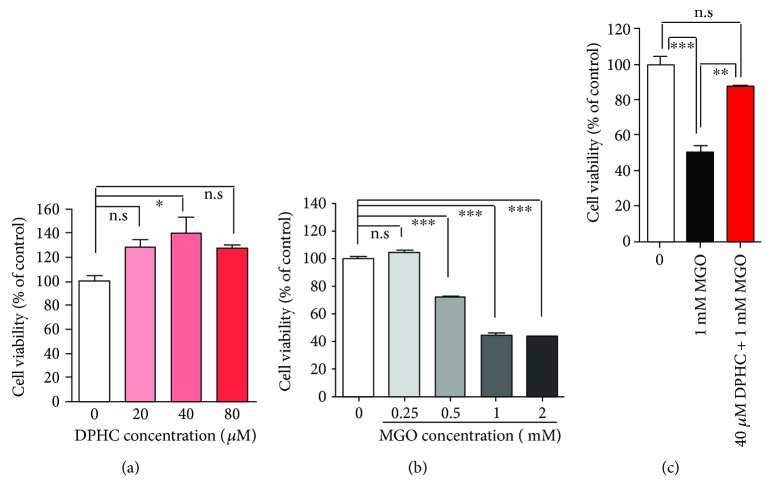
DPHC attenuates MGO-induced toxicity in HEK cells. (a) HEK cells were incubated with the indicated concentrations of DPHC for 24 h. (b) HEK cells were incubated with the indicated concentrations of MGO for 24 h. (c) HEK cells were incubated with or without 40 *μ*M DPHC for 1 h and then further incubated with 1 mM MGO for 24 h. CCK-8 assays were subsequently performed. Experiments were performed in triplicate. ^∗^*p* < 0.05, ^∗∗^*p* < 0.01, and ^∗∗∗^*p* < 0.001. n.s.: no significance.

In order to determine whether DPHC has a protective effect on MGO-induced toxicity in human kidney cells, we treated HEK (human embryonic kidney) cells with MGO. DPHC alone did not show any cytotoxicity in cells, even at a high concentration (80 *μ*M) (Figure 2(a)), whereas MGO showed cytotoxicity in a dose-dependent manner (Figure 2(b)). 40 *μ*M of DPHC used for the following experiments as it induced the highest cell proliferation, and 1 mM MGO concentration was used as it showed 50% lethal concentration. Pretreatment with 40 *μ*M DPHC significantly blocked the cytotoxic effect of MGO (Figure 2(c)), indicating that DPHC possesses a protective effect against MGO-induced toxicity in human kidney cells.

### 3.2. DPHC Scavenges MGO-Induced ROS

**Figure 3:**
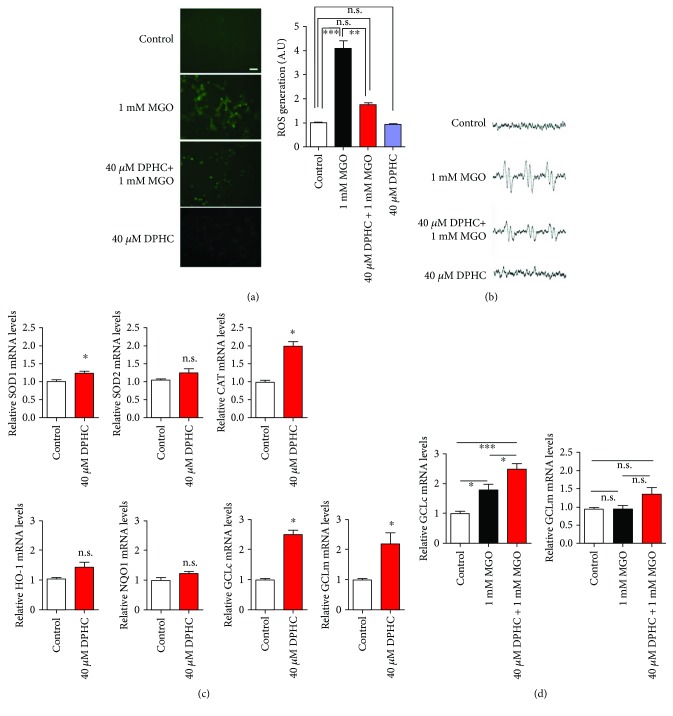
DPHC scavenges MGO-induced ROS in HEK cells. HEK cells were incubated with or without 40 *μ*M DPHC for 1 h and then further incubated with or without 1 mM MGO for 24 h. (a) Intracellular ROS was measured using an oxidation-sensitive fluorescent probe dye (left panel) and quantified (right panel). Scale bar: 200 *μ*m. (b) Alkyl radical scavenging was measured using ESR spectra. (c) HEK cells were incubated with 40 *μ*M DPHC for 24 h. qRT-PCR was performed for superoxide dismutase (SOD) 1 and 2, catalase (CAT), heme oxygenase-1 (HO-1), NAD(P)H quinone dehydrogenase (NQO)1, glutamylcysteine synthetase (GCL)c, and GCLm. (d) HEK cells were incubated with or without 40 *μ*M DPHC for 1 h and then further incubated with 1 mM MGO for 24 h. qRT-PCR was performed for GCLc and GCLm. ^∗^*p* < 0.05, ^∗∗^*p* < 0.01, and ^∗∗∗^*p* < 0.001. n.s.: no significance.

DPHC shows powerful radical scavenging activity [20]. An excess production of MGO can increase ROS [23]. So, we determined whether DPHC can attenuate the production of MGO-induced ROS. As expected, ROS production was significantly increased by MGO treatment, whereas pretreatment with DPHC significantly reduced MGO-induced ROS (Figure 3(a)). Additionally, the intracellular alkyl radical spectrum was elevated over controls by MGO treatment, whereas DPHC pretreatment of cells reduced this elevation (Figure 3(b)). DPHC alone did not affect ROS production or the alkyl radical spectrum (Figures 3(a) and 3(b)).

ROS scavenging effects involve antioxidant activity and detoxification. So, we determined the effect of DPHC treatment on antioxidant and phase II detoxifying enzyme expression. mRNA levels of the antioxidant enzymes superoxide dismutase (SOD)1 and catalase (CAT) and the phase II detoxifying enzymes *γ*-glutamylcysteine synthetase (GCL)c and GCLm were significantly increased in DPHC-treated cells. Mean levels of SOD2, heme oxygenase- (HO-) 1, and NAD(P)H quinone dehydrogenase (NQO)1 were elevated over the controls, but did not reach significance (Figure 3(c)). When we measured mRNA expression in DPHC-pretreated cells given subsequent MGO treatment, we found that mRNA expression of antioxidant enzymes (SOD, CAT, and HO-1) and NQO-1 was increased by MGO treatment and was not significantly changed compared with MGO treatment only (data not shown). The GCLc mRNA expression level was increased by MGO treatment and further increased in DPHC-pretreated cells given MGO treatment (Figure 3(d)). These results indicate that DPHC attenuates MGO induction through *γ*-glutamylcysteine synthetase detoxifying enzyme activation.

### 3.3. DPHC Activates Nrf2 Transcription Factor

**Figure 4:**
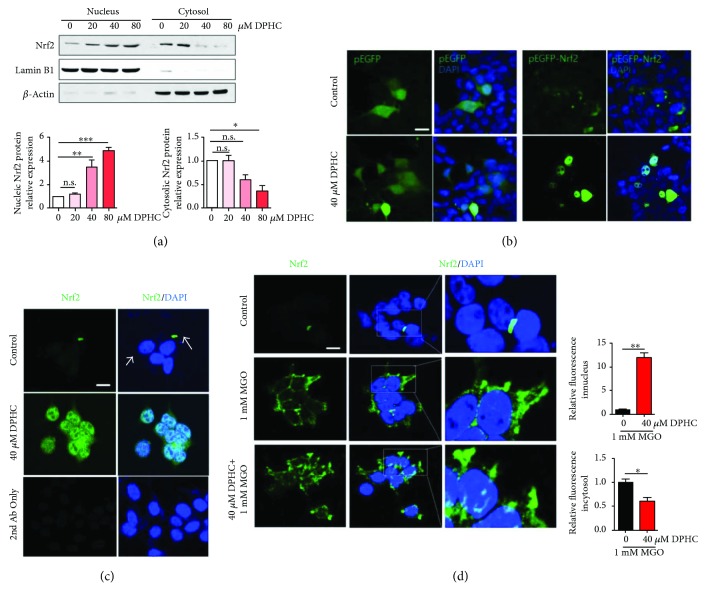
DPHC activates Nrf2 transcription factor in HEK cells. (a) HEK cells were incubated with the indicated concentrations of DPHC for 24 h. Nuclear and cytoplasmic fractions were prepared, and Western blotting was subsequently performed. (b) HEK cells were transfected with plasmids for EGFP-tagged Nrf2 or EGFP for 33 h and further incubated with or without DPHC (40 *μ*M) for 24 h. The cells were then fixed and observed by confocal microscopy. Scale bar indicates 10 *μ*m. (c) HEK cells were incubated with or without DPHC (40 *μ*M) for 24 h. The cells were stained with an anti-Nrf2 (green) antibody and observed using confocal microscopy. Blue indicates DAPI (nuclear) staining. Scale bar indicates 10 *μ*m. (d) HEK cells were incubated with or without DPHC (40 *μ*M) for 1 h and then further incubated with 1 mM MGO for 6 h. The cells were stained with an anti-Nrf2 (green) antibody and observed using confocal microscopy. Blue indicates DAPI (nuclear) staining. Scale bar indicates 10 *μ*m. ^∗^*p* < 0.05, ^∗∗^*p* < 0.01, and ^∗∗∗^*p* < 0.001. n.s.: no significance.

DPHC is a polyphenolic compound that targets Nrf2 genes [24], and Nrf2 is well known as a master regulator of antioxidants and phase II detoxification enzymes [25]. So, we determined whether DPHC modulates Nrf2 signaling. The expression level of Nrf2 protein in the nuclear fraction was increased in a dose-dependent manner by treatment with DPHC (Figure 4(a)). The expression of transfected Nrf2-EGFP (Figure 4(b)) and antibody staining of endogenous Nrf2 (Figure 4(c)) was higher in DPHC-treated HEK cells as compared with controls. Furthermore, Nrf2 expression was increased particularly in cytosol by MGO treatment, and this was decreased by pretreatment with DPHC. In addition, Nrf2 appeared to translocate to the nucleus as a result of DPHC pretreatment (Figure 4(d)).

**Figure 5:**
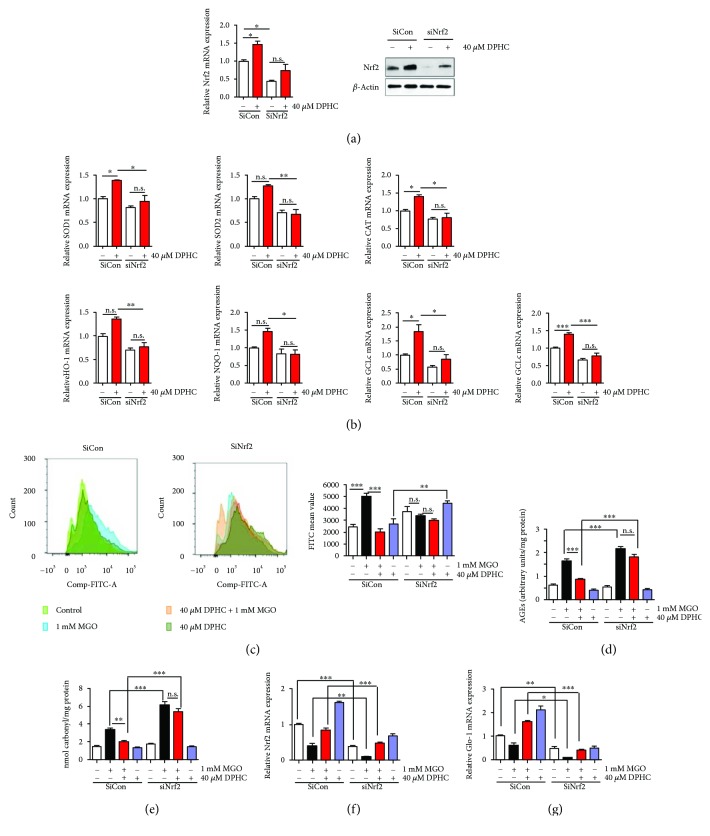
DPHC did not affect antioxidant, detoxifying, ROS, AGEs, or protein carbonyl content in Nrf2-knockdown HEK cells. HEK cells were transfected with Nrf2 siRNA for 36 h. The transfected cells were incubated with 40 *μ*M DPHC for 24 h. (a) qRT-PCR and Western blot were performed for nuclear factor (erythroid-derived 2)-like 2 (Nrf2) mRNA and protein expression. (b) qRT-PCR was performed for superoxide dismutase (SOD) 1 and 2, catalase (CAT), heme oxygenase-1 (HO-1), NAD(P)H quinone dehydrogenase (NQO)1, glutamylcysteine synthetase (GCL)c, and GCLm. (c–g) HEK cells were transfected with Nrf2 siRNA for 36 h. The transfected cells were incubated with or without 40 *μ*M DPHC for 1 h and then further incubated with or without 1 mM MGO for 24 h. (c) ROS production was measured by flow cytometry. (d) AGE content was measured. (e) Protein carbonyl content was measured. (f) Nrf2 mRNA expression was measured by qRT-PCR. (g) Glo-1 (glyoxalase 1) mRNA expression was measured by qRT-PCR. Experiments were performed in triplicate. ^∗^*p* < 0.05, ^∗∗^*p* < 0.01, and ^∗∗∗^*p* < 0.001. n.s.: no significance.

To confirm whether the effect of DPHC is mediated through Nrf2 signaling, we downregulated Nrf2 using siRNA. Transfection of Nrf2 siRNA significantly downregulated both Nrf2 mRNA and protein expression (Figure 5(a)). mRNA expression of antioxidant enzymes including SOD1 and CAT and phase II detoxification enzymes including GCLc and GCLm was increased in control siRNA-transfected cells by DPHC treatment; however, this increase was inhibited in Nrf2 siRNA-transfected cells (Figure 5(b)). ROS production was increased by MGO treatment, and MGO-induced ROS production was inhibited by DPHC pretreatment in the control siRNA-transfected cells. In the Nrf2 knockdown cells, DPHC pretreatment did not inhibit MGO-induced ROS production (Figure 5(c)). In addition, DPHC pretreatment did not inhibit AGE and protein carbonyl production induced by MGO treatment in Nrf2 siRNA-transfected cells (Figures 5(d) and 5(e)). mRNA expression of Nrf2 and Glo-1 was decreased by MGO treatment, and the decreased Nrf2 and Glo-1 mRNA expression was recovered by DPHC pretreatment in the control siRNA-transfected cells. In the Nrf2 knockdown cells, the effect of DPHC treatment on the recovery of Glo-1 mRNA expression was significantly less compared with that in the control siRNA-transfected cells. Thus, the changes in Nrf2 expression were positively correlated with the changes in Glo-1 mRNA expression (Figures 5(f) and 5(g)). These results suggest that DPHC induces antioxidant as well as detoxifying properties by activating Nrf2.

### 3.4. DPHC Protects against MGO-Induced Glycation

**Figure 6:**
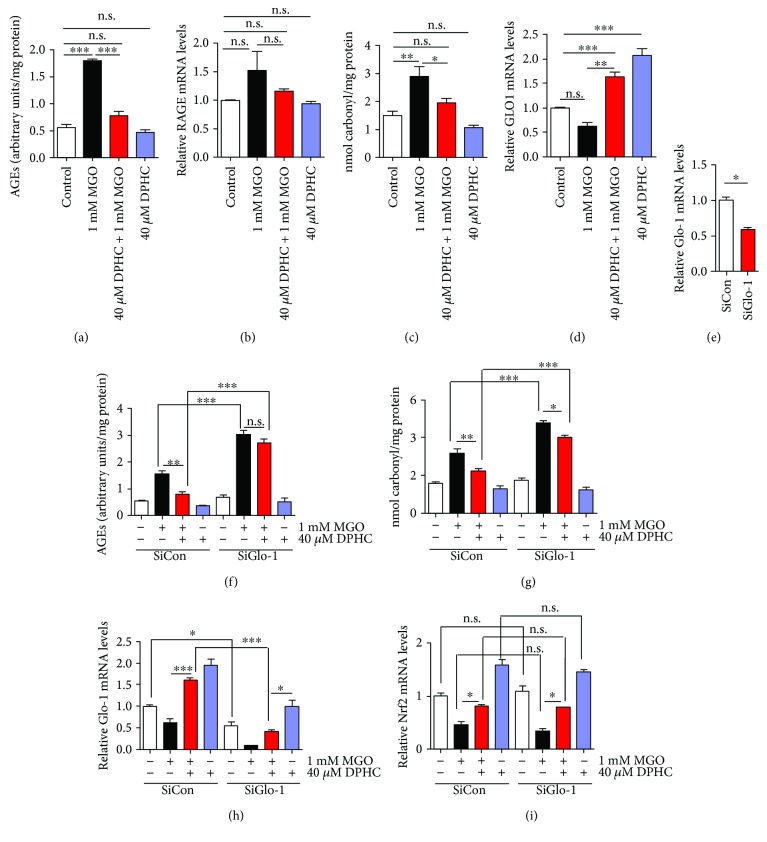
DPHC protects MGO-induced protein glycation in HEK cells. HEK cells were incubated with or without 40 *μ*M DPHC for 1 h and then further incubated with or without 1 mM MGO for 24 h. (a) AGE content was measured. (b) AGE receptor (RAGE) mRNA expression level was analyzed by qRT-PCR. (c) Protein carbonyl content was measured. (d) Glo-1 mRNA expression level was analyzed by qRT-PCR. (e–i) HEK cells were transfected with Glo-1 siRNA for 36 h and incubated with or without 40 *μ*M DPHC for 1 h and then further incubated with or without 1 mM MGO for 24 h. (e) Glo-1 mRNA expression level was analyzed by qRT-PCR. (f) AGE content was measured. (g) Protein carbonyl content was measured. (h) Glo-1 (glyoxalase 1) expression was measured by qRT-PCR. (i) Nuclear factor (erythroid-derived 2)-like 2 (Nrf2) mRNA expression was measured by qRT-PCR. Experiments were performed in triplicate. ^∗^*p* < 0.05, ^∗∗^*p* < 0.01, ^∗∗∗^*p* < 0.001. n.s. indicates no significance.

AGE formation is a critical factor in diabetic complications; particularly, the accumulation of MGO-derived AGEs has been implicated in DN [26]. Therefore, we determined whether DPHC alleviates AGE formation caused by MGO in human kidney cells. AGE formation was significantly increased in MGO-treated cells, whereas pretreatment with DPHC prevented MGO-induced AGE formation (Figure 6(a)). AGEs exert their harmful effects directly or indirectly through their interaction with the receptor for AGEs (RAGE) [27, 28]. Therefore, we also analyzed the mRNA expression level of RAGE. RAGE mRNA levels were not significantly different from controls either in MGO-treated cells or in MGO-treated cells pretreated with DPHC (Figure 6(b)). MGO is oxidatively modified to form carbonyl proteins [29, 30]. Therefore, we determined whether DPHC attenuates protein carbonyl formation caused by MGO. The protein carbonyl content was significantly elevated in MGO-treated cells, whereas pretreatment with DPHC prevented MGO-induced protein carbonyl formation (Figure 6(c)).

Glyoxalase-1 (Glo-1) is an enzyme in a metabolic pathway that detoxifies *α*-oxoaldehydes, particularly MGO [31]. Glo-1 mRNA levels were increased in DPHC-pretreated cells (Figure 6(d)). On the other hand, AGEs, RAGE, and protein carbonyls were not altered by DPHC alone. To confirm whether the glycation-attenuating effect of DPHC is mediated by Glo-1 expression, we downregulated Glo-1 using siRNA. Transfection of Glo-1 siRNA significantly downregulated Glo-1 mRNA expression (Figure 6(e)). The AGE content and protein carbonyl content induced by MGO were reduced by DPHC pretreatment in control siRNA-transfected cells; however, these effects were abolished in the Glo-1-downregulated cells (Figures 6(f) and 6(g)). As expected, Glo-1mRNA expression was decreased by MGO treatment, and this decrease was recovered by DPHC pretreatment in the control siRNA-transfected cells. However, DPHC pretreatment did not increase Glo-1 mRNA expression in the Glo-1 knockdown cells (Figure 6(h)). mRNA expression of Nrf2 was also decreased by MGO treatment, and the decreased Nrf2 mRNA expression was recovered by DPHC pretreatment in the control siRNA-transfected cells. Glo-1 knockdown did not change these effects (Figure 6(i)). These results suggest that DPHC induces a protective effect against MGO-induced protein glycation by increasing the expression of Glo-1 mRNA.

### 3.5. DPHC Prevents AGEs and Protein Carbonyl Production in a Hyperglycemic Condition

**Figure 7:**
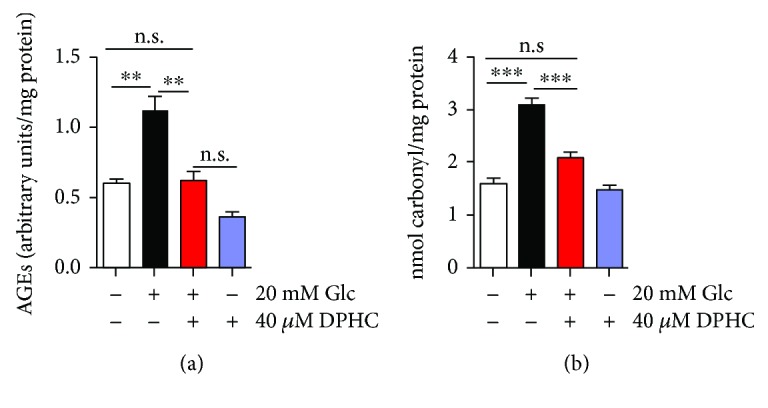
DPHC prevents AGE and protein carbonyl production in hyperglycemic HEK cells. HEK cells were incubated with 40 *μ*M DPHC for 1 h and then further incubated with or without 20 mM glucose (Glc) for 30 h. (a) AGE content was measured. (b) Protein carbonyl content was measured. Experiments were performed in triplicate. ^∗∗^*p* < 0.01 and ^∗∗∗^*p* < 0.001. n.s.: no significance.

The hyperglycemic environment can cause major health complications in people with diabetes [32], and DN does not develop in the absence of hyperglycemia [33]. Therefore, we examined the effect of high glucose on AGEs and protein carbonyls. The AGE content was significantly elevated in glucose-treated cells, whereas pretreatment with DPHC prevented this rise (Figure 7(a)). The protein carbonyl content was also significantly elevated in the glucose-treated cells, whereas pretreatment with DPHC prevented glucose-induced protein carbonyl formation (Figure 7(b)). DPHC alone did not affect either AGE or protein carbonyl formation. These results suggest that DPHC attenuates glycation caused by hyperglycemia.

## 4. Discussion

Recent evidence indicates that reactive carbonyls including MGO cause diabetic complications such as DN [34–36]. Reactive carbonyls are responsible for AGE formation, and AGE accumulation is observed in most DN patients, which is a major factor in the pathogenesis of DN [35–37] due to AGE degradation dysfunction in kidney [38]. Therefore, clearance of accumulated AGE or inhibition of AGE formation is extremely important in treating DN.

Natural products such as polyphenols have been used as an alternative treatment for diabetes [39–44] and diabetes complications [45] in many countries. Polyphenolic compounds which are known to show antioxidant effects are found in the edible seaweed *Ishige okamurae* (Table 2). However, it is not known whether DHPC, one of polyphenolic compounds from the *I. okamurae*, might ameliorate diabetic complications, particularly DN. In the present study, we investigated the protective effects of DPHC on MGO-induced AGE formation in HEK cells.

Our study demonstrated that MGO treatment caused cytotoxicity, increased ROS, and increased AGE and carbonyl protein formation in HEK cells. All of these effects were significantly attenuated by pretreatment with DPHC. Oxidative stress is one of the primary phenomena leading to renal dysfunction [46, 47]. Increased ROS has also been observed in end-stage renal disease [48], implying that reduced antioxidant potential may be involved in the oxidative stress related to DN. Although most cells have mechanisms to protect themselves against toxic stimuli such as oxidative stress, unwanted by-products can overwhelm the natural antioxidative defense system. Thus, dietary supplements containing antioxidants may be important for additional protection against oxidative stress and the prevention of DM.

Our results showed that the expression of antioxidant and phase II detoxifying enzymes including SOD1, CAT, GCLc, and GCLm was increased after incubation with DPHC. These results suggest that DPHC exerts its protective effects via induction in the expression of antioxidant enzymes, particularly through *γ*-glutamylcysteine synthetase-detoxifying enzyme activation. Similarly, other studies have reported that DPHC increases the levels of antioxidant enzymes, including SOD, CAT, and glutathione peroxidase [49], and reduces the levels of proinflammatory enzymes, including nitric oxide synthase and cyclooxygenase-2, thus inhibiting ROS formation, or by blocking ROS-induced apoptotic pathways [15, 50].

Cellular removal of reactive carbonyls and AGEs occurs via the glutathione-dependent Glo-1 enzyme, and Glo-1 is found in all tissues, including renal tissue [51]. In addition, Glo-1 overexpression reduces hyperglycemia-induced expression of reactive carbonyl stress, AGEs, and oxidative stress [52, 53]. Knockdown of Glo-1 expression in nondiabetic mice by siRNA results in the development of albuminuria and mesangial expansion [54]. We found that treatment with DPHC increased expression of Glo-1, and Glo-1 knockdown abolished the inhibitory effects of DPHC pretreatment on MGO-induced AGE production and protein carbonylation, suggesting that this detoxification pathway is one mechanism for the protective effect of DPHC.

The transcription factor Nrf2 plays a major role in the regulation of redox homeostasis and the cellular detoxification response [55–57] by modulating the gene expression of a number of enzymes that detoxify prooxidative stressors [58]. Nrf2 activation reduces ROS overproduction caused by AGEs [59, 60] and thus can be a therapeutic target for diabetic complications [61, 62]. In addition, reactive carbonyl stress is countered by upregulation of Glo-1 by the Nrf2 signaling pathway, which protects proteins and DNA from increased damage and preserves cell function [63]. Polyphenols from natural products exert their health effects by activating the Nrf2 signaling system [24, 64, 65]. In this study, we found that DPHC activated Nrf2, suggesting that this may be a mechanism by which antioxidant enzymes and Glo-1 are increased. Our results show that DPHC pretreatment significantly decreased MGO-induced AGE accumulation. Therefore, reduction of AGE accumulation by DPHC is probably due to the inhibition of AGE formation by induction of Glo-1 expression. Natural phenolic compounds also show antiglycation effects, and the molecular mechanisms underlying this action are trapping of MGO, activation of Glo-1, inhibition of AGE formation, and blockage of RAGE in addition to their antioxidant effects [66]. DPHC treatment did not affect the expression of RAGE mRNA, suggesting that a decrease in the accumulated amount of AGE might contribute to the preventive effects on MGO-induced cytotoxicity. In agreement with this result, it was reported that circulating and tissue levels of AGE-modified proteins were increased in diabetic db/db mice, but RAGE expression levels in the renal cortex were not different between diabetic and nondiabetic littermates [67].

**Figure 8:**
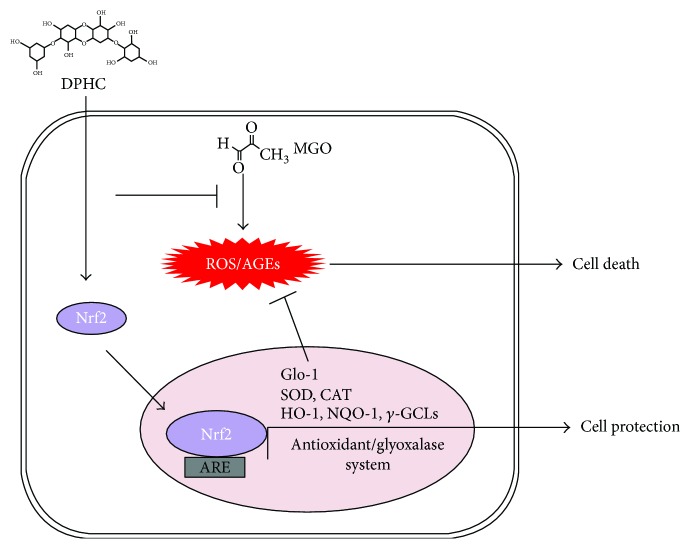
Possible mechanisms for the inhibition of AGE accumulation by DPHC in MGO-treated HEK cells. DPHC promotes the expression of not only antioxidant and phase II detoxifying enzymes but also Glo-1 by mediating Nrf2 activation. This inhibits oxidative stress and formation of AGEs, effectively attenuating cell damage. DPHC: diphlorethohydroxycarmalol; MGO: methylglyoxal; Nrf2: nuclear factor erythroid 2-related factor 2; ARE: antioxidant response element; ROS: reactive oxygen species; AGEs: advanced glycation end products; Glo-1: glyoxalase 1; SOD: superoxide dismutase; CAT: catalase; HO-1: heme oxygenase-1; NQO: NAD(P)H quinone dehydrogenase 1; GCL: *γ*-glutamylcysteine synthetases.

Accordingly, DPHC may reduce AGE accumulation via a variety of mechanisms, including Nrf2 transcription activation, in addition to its antioxidant effects (Figure 8). We suggest that DPHC can be used not only as an easily accessible source of natural antioxidants but also as an ingredient for functional food and pharmaceutical agents related to DN.

## Figures and Tables

**Table 1:** Primer sequences.

Gene name	Sequence 5′-3′	
SOD1	Forward	5′-GGT CCT CAC TTT AAT CCT CTA T-3′
	Reverse	5′-CAT CTT TGT CAG CAG TCA CAT T-3′
SOD2	Forward	5′-TTC TGG ACA AAC CTC AGC CC-3′
	Reverse	5′-AGT TTG ATG GCT TCC AGC A-3′
CAT	Forward	5′-TTT CCC AGG AAG ATC CTG AC-3′
	Reverse	5′-ACC TTG GTG AGA TCG AAT GG-3′
GPx	Forward	5′-AGA ATG TGG CGT CCC TCT GA-3′
	Reverse	5′-CAG CTC GTT CAT CTG GGT GTA G-3′
HO-1	Forward	5′-CAG GCA ATG GCC TAA ACT TC-3′
	Reverse	5′-GCT GCC ACA TTA GGG TGT CT-3′
NQO1	Forward	5′-GTT GCC TGA AAA ATG GGA GA-3′
	Reverse	5′-AAA AAC CAC CAG TGC CAG TC-3′
GCLc	Forward	5′-AGT TGA GGC CAA CAT GCG AA-3′
	Reverse	5′-TGA AGC GAG GGT GCT TGT TT-3′
GCLm	Forward	5′-ATC AAA CTC TTC ATC ATC AAC-3′
	Reverse	5′-GAT TAA CTC CAT CTT CAA TAG G-3′
Glo-1	Forward	5′-ATGCGACCCAGAGTTACCAC-3′
	Reverse	5′-CCAGGCCTTTCATTTTACCA-3′
RAGE	Forward	5′-GTGGGGACATGTGTGTCAGAGGGAA-3′
	Reverse	5′-TGAGGAGAGGGCTG GGCAGGGACT-3′
Cyclophilin	Forward	5′-TGC CAT CGC CAA GGA GTA G-3′
	Reverse	5′-TGC ACA GAC GGT CAC TCA AA-3′

**Table 2:** Polyphenolic compounds isolated from *Ishige okamurae.*

Name	Structure	Function
Phloroglucinol	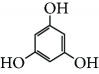	Antioxidant [68]
6,6′-Bieckol	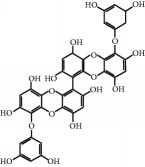	Acetylcholinesterase (AChE) inhibitory effect [69]; antioxidant [68]
Diphlorethohydroxycarmalol (DPHC)	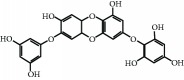	Butyrylcholinesterase (BChE) inhibitory activity [69]; antioxidant [68]; antiobesity [19]; antidiabetic
